# 

**Published:** 1907-02-15

**Authors:** 


					﻿Dental
Register

NELVILjLE S. HQFF, Editor,
______ /	Ann Arbor, IlVUch.
SAMUEL A. CROCKER & CO., Publishers
OHIO DENTAL AND SURGICAL DEPOT
35 W. FIFTH STREET, CINCINNATI, 0.
PRICE, ONE DOLLAR A YEAR
				

